# Emergency Colorectal Cancer During COVID-19: A Retrospective Cohort Study of Temporal Trends in Presentation, Tumour Characteristics, and Management in a Regional Australian Health Service

**DOI:** 10.7759/cureus.117364

**Published:** 2026-10-02

**Authors:** Olivia McNeil, Marcio B Coehlo, Zachary Woodward, Bree Stephensen

**Affiliations:** 1 General Surgery, Sunshine Coast University Hospital, Sunshine Coast, AUS

**Keywords:** colorectal cancer (crc), covid-19, emergency presentation, healthcare disruption, regional health service, stoma formation, tumour grade

## Abstract

Introduction

COVID-19 disrupted colorectal cancer (CRC) screening and diagnostic services, raising concerns that delayed diagnosis would increase emergency presentations and advanced disease. This study evaluated temporal changes in emergency CRC presentation, tumour characteristics, and management within a regional Australian health service.

Methods

A retrospective cohort study included adults presenting emergently with newly diagnosed CRC between January 2019 and July 2023. Absolute emergency presentation volumes and emergency presentations relative to elective CRC resections were examined before and during COVID-19 restrictions. Temporal trends in tumour characteristics and management were assessed using logistic regression, with secondary analyses adjusted for age and sex.

Results

A total of 149 patients presented emergently with CRC. Absolute emergency presentation volumes remained stable across the complete calendar years studied, with 30 cases in 2019, 28 in 2020, 32 in 2021, and 30 in 2022. Emergency presentations also remained stable relative to elective CRC resections, accounting for 15.3% of CRC surgical activity in 2019 compared with 15.2% during the COVID-19 restriction period (2020-2022; odds ratio (OR) 0.99, 95% confidence interval (CI) 0.63-1.55; p = 0.970). Increasing calendar year was independently associated with greater odds of high-grade disease (adjusted OR (aOR) 1.39 per year, 95% CI 1.07-1.82; p = 0.015) and stoma formation at index presentation (aOR 1.37 per year, 95% CI 1.04-1.80; p = 0.026), although the latter association was influenced by a marked increase in stoma formation in 2022. Stage IV and metastatic disease were more frequent in later years but did not demonstrate statistically significant temporal associations.

Conclusion

Emergency CRC presentation volumes remained stable during the COVID-19 restriction period within this regional Australian health service, alongside relatively preserved elective CRC surgical activity. Although increasing calendar year was associated with high-grade disease and stoma formation, the stoma association was influenced by a marked increase in 2022 and should be interpreted cautiously. These findings suggest that presentation frequency alone may not capture changes in the characteristics and management complexity of emergency CRC.

## Introduction

The COVID-19 pandemic caused substantial disruption to cancer screening, diagnostic services, and elective healthcare worldwide [1,2]. Reductions in population-based screening and access to endoscopic investigation raised concerns that delayed colorectal cancer (CRC) diagnosis could result in more patients presenting emergently with advanced disease. The extent of this disruption varied considerably between countries and Australian jurisdictions, making evaluation of local health-system outcomes important when assessing the impact of the pandemic.

CRC is among the most commonly diagnosed malignancies worldwide, and early detection through screening facilitates diagnosis at an earlier stage and planned elective treatment. Emergency CRC may present with bowel obstruction, perforation, or significant bleeding and is associated with greater postoperative morbidity and mortality than elective surgery [3,4]. Understanding changes in emergency presentation may therefore provide insight into the downstream effects of disruption to cancer diagnostic and treatment pathways.

In Australia, five-year relative survival from CRC has improved substantially over recent decades alongside advances in screening and treatment [5]. The National Bowel Cancer Screening Program (NBCSP) provides population-based screening using an immunochemical faecal occult blood test (iFOBT), offered during the study period to eligible Australians aged 50-74 years, with participants who return a positive result referred for further diagnostic assessment, typically colonoscopy. Between January and September 2020, colonoscopy and sigmoidoscopy activity declined by approximately 15% compared with the corresponding period in 2019, representing more than 78,000 fewer procedures nationally [6]. International studies similarly reported disruption to CRC diagnosis and treatment, raising concerns regarding delayed diagnosis, emergency presentation, and more advanced disease [1,3,7-9].

Within our health service, clinicians perceived an increase in patients presenting emergently with complicated or advanced CRC during the pandemic. Although broader Australasian data have described disruption to CRC care [2], the effect on emergency presentation patterns within regional Australian health services remains incompletely characterised. Furthermore, assessment of presentation frequency alone may not identify changes in the tumour characteristics or management complexity of patients who present emergently. Understanding these effects may also inform efforts to preserve cancer diagnostic pathways during future periods of healthcare disruption.

This study therefore aimed to evaluate emergency CRC presentations within a regional Queensland health service between January 2019 and July 2023, encompassing pre-pandemic, COVID-19 restriction, and early post-restriction periods. The primary objective was to assess changes in the absolute volume of emergency CRC presentations and determine whether emergency presentations relative to elective CRC surgical activity increased during the COVID-19 restriction period. Secondary objectives were to evaluate temporal changes in clinical presentation, tumour stage and grade, metastatic disease, and management, and to describe documented prior engagement with colonoscopy and the NBCSP.

## Materials and methods

Study design and setting

This retrospective observational cohort study was conducted within Sunshine Coast Hospital and Health Service (SCHHS), Queensland, Australia. SCHHS is a regional health service providing colorectal surgical services to a mixed urban and regional population. The study period extended from January 1, 2019, to July 31, 2023, and encompassed the pre-pandemic, COVID-19 restriction, and early post-restriction periods in Queensland. For descriptive purposes, study periods were defined as pre-pandemic (2019), COVID-19 restriction period (2020-2022), and early post-restriction period (January-July 2023). The 2020-2022 period was grouped for comparison with the pre-pandemic period, whilst annual data were also examined separately to assess temporal variation within the pandemic period.

Study population

All adult patients presenting emergently with previously undiagnosed CRC during the study period were included. Emergency presentation was defined as an index acute hospital presentation resulting in the diagnosis of CRC, including presentations with bowel obstruction, perforation, significant gastrointestinal bleeding, or other acute complications requiring urgent assessment or intervention.

Patients were included irrespective of subsequent management strategy. This included patients undergoing emergency tumour resection with primary anastomosis or stoma formation, non-resectional diverting or palliative stoma formation, endoscopic colonic stenting, and non-operative management.

Annual numbers of emergency CRC presentations were examined directly. For analysis of emergency CRC presentation relative to elective surgical activity, annual numbers of elective CRC resections performed within SCHHS were obtained from institutional surgical records to provide a comparator denominator. Patients undergoing elective CRC resection without a preceding emergency presentation were not included in the detailed patient-level clinical, pathological, or management analyses.

Data collection

Patient demographics, mode of presentation, tumour characteristics, staging, metastatic disease, management strategy, and documented screening history were extracted retrospectively from the electronic medical record, operative reports, endoscopy records, and colorectal unit datasets. Tumour stage was recorded according to the TNM classification. Pathological staging was used where available following tumour resection. For patients managed without resection, clinical stage was determined from available radiological, endoscopic, and multidisciplinary assessment. Patients with radiologically demonstrated distant metastatic disease were classified as clinical stage IV irrespective of whether pathological staging was available. Where patients did not undergo sufficient staging investigations or surgical resection to permit reliable assignment of tumour stage, stage was recorded as unknown. This included patients managed with palliative intent in whom further investigation or treatment was not pursued because of overall clinical circumstances, including comorbidity, rather than necessarily because of known advanced or metastatic disease. Metastatic disease at presentation was recorded separately and determined from available radiological, pathological, and multidisciplinary assessment at the index presentation. Where available investigations were insufficient to reliably determine the presence or absence of distant metastatic disease, metastatic status was recorded as unknown.

Tumour grade was categorised as low, moderate, or high. Undifferentiated tumours were classified as high grade. For analysis of temporal trends, low- and moderate-grade tumours were grouped and compared with high-grade tumours.

Prior colonoscopy and participation in the NBCSP were determined from available medical record documentation. Where these or other variables were not documented or could not be established, they were recorded as unknown.

Outcomes

The primary outcomes were the absolute annual volume of emergency CRC presentations and the proportion of combined emergency CRC presentations and elective CRC resections represented by emergency presentations during the COVID-19 restriction period (2020-2022) compared with the pre-pandemic period (2019). Secondary outcomes included mode of emergency presentation, tumour grade, stage at presentation, presence of metastatic disease, management strategy, stoma formation during the index emergency presentation, and documented prior colonoscopy or NBCSP participation.

Management strategy was classified as resection with primary anastomosis, resection with stoma formation, non-resectional stoma formation, colonic stenting, or non-operative management. Stoma formation at index presentation was defined as formation of any stoma during management of the initial emergency presentation, whether performed in association with tumour resection or as a non-resectional diverting or palliative procedure.

Statistical analysis

Descriptive statistics were used to summarise clinical, pathological, and management characteristics. Categorical variables were reported as frequencies and percentages. Absolute annual emergency CRC presentation volumes were reported descriptively. Where variables contained missing or unknown data, percentages were calculated using the available-case denominator; missing values were not imputed. Patients with unknown tumour stage or metastatic status were excluded only from analyses requiring the respective variable and remained included in analyses of other outcomes where the relevant data were available.

The proportion of combined emergency CRC presentations and elective CRC resections represented by emergency presentations during the COVID-19 restriction period (2020-2022) was compared with the pre-pandemic period (2019) using logistic regression, with results reported as odds ratios (ORs) and 95% confidence intervals (CIs).

Temporal trends in emergency presentation and selected secondary outcomes were evaluated using logistic regression with calendar year modelled as a continuous variable. Selected secondary outcomes included stage IV disease, high-grade tumour, metastatic disease at presentation, primary anastomosis, and stoma formation at the index emergency presentation. ORs therefore represent the change in odds of each outcome associated with each successive calendar year.

Multivariable logistic regression models were additionally fitted for these secondary outcomes, adjusting for age and sex. Results are reported as adjusted ORs (aORs) with 95% CIs. Overall differences in categorical clinical presentation and management strategy across study years were assessed using chi-squared or Fisher's exact tests, as appropriate.

Secondary analyses were considered exploratory. Given the retrospective study design and modest sample size, findings were interpreted with consideration of effect estimates and CIs in addition to statistical significance. All statistical tests were two-sided, and a p-value < 0.05 was considered statistically significant.

Ethical considerations

This study received ethical approval from the Metro North Health Human Research Ethics Committee (HREC/2023/MNHA/99476), with site-specific governance authorisation from SCHHS (SSA/2024/QSC/99476). A waiver of individual patient consent was approved by the HREC. All data were de-identified prior to analysis and handled in accordance with institutional governance requirements.

## Results

Emergency CRC presentations over time

Between January 2019 and July 2023, 149 patients presented emergently with CRC within SCHHS. Annual emergency presentation numbers were 30 in 2019, 28 in 2020, 32 in 2021, 30 in 2022, and 29 between January and July 2023 (Table 1). Corresponding elective CRC resections numbered 166, 182, 167, 154, and 117, respectively. Absolute emergency presentation volumes therefore remained stable across the four complete calendar years from 2019 to 2022, ranging from 28 to 32 presentations annually.

**Table 1:** Emergency colorectal cancer (CRC) presentations and elective CRC resections by year. The 2023 data include January-July only. The COVID-19 era comparison comprises calendar years 2020-2022. Combined total represents emergency CRC presentations plus elective CRC resections.

Year	Emergency CRC presentations, n	Elective CRC resections, n	Combined total, n	Emergency proportion, %
2019	30	166	196	15.3
2020	28	182	210	13.3
2021	32	167	199	16.1
2022	30	154	184	16.3
2020-2022 (COVID-19 era)	90	503	593	15.2
2023 (Jan-July)	29	117	146	19.9

Emergency presentation proportions also remained relatively stable during the pre-pandemic and COVID-19 restriction periods. Using the combined number of emergency CRC presentations and elective CRC resections as the denominator, emergency presentations accounted for 15.3% (30/196) in 2019 and 15.2% (90/593) during 2020-2022 (OR 0.99, 95% CI 0.63-1.55; p = 0.970). There was no significant difference across individual study years (p = 0.588), nor evidence of a significant linear temporal trend (OR 1.09 per year, 95% CI 0.96-1.24; p = 0.190). Between January and July 2023, the emergency presentation proportion was 19.9% (29/146).

Clinical presentation and tumour characteristics

The distribution of clinical presentation did not differ significantly across study years (p = 0.423) (Table 2). Bowel obstruction was the most frequent mode of presentation throughout the study period, accounting for 48.3%-60.7% of presentations annually. Perforation was uncommon, whilst bleeding presentations varied from 9.4% in 2021 to 37.9% in 2023.

**Table 2:** Clinical and pathological characteristics of emergency colorectal cancer presentations by year. Data are presented as n (%) unless otherwise specified. *2023 includes January-July only. ^†^Overall p-values compare distributions across calendar years. Temporal trends in selected binary outcomes are reported separately in Table 3. ^‡^Percentages calculated among patients with available data. Tumour stage and metastatic status were known in 144/149 patients; five patients had insufficient staging investigations to permit reliable determination of tumour stage or metastatic status. Tumour grade was known in 127/149 patients. Undifferentiated tumours were classified as high grade.

Characteristic	2019 n = 30	2020 n = 28	2021 n = 32	2022 n = 30	2023* n = 29	p-value^†^
Clinical presentation						0.423
Obstruction	17 (56.7)	17 (60.7)	18 (56.3)	16 (53.3)	14 (48.3)	
Perforation	3 (10.0)	1 (3.6)	1 (3.1)	2 (6.7)	0	
Bleeding	5 (16.7)	7 (25.0)	3 (9.4)	6 (20.0)	11 (37.9)	
Other	5 (16.7)	3 (10.7)	10 (31.3)	6 (20.0)	4 (13.8)	
Stage IV disease^‡^	12/29 (41.4)	9/27 (33.3)	12/29 (41.4)	17/30 (56.7)	15/29 (51.7)	
High-grade tumour^‡^	7/24 (29.2)	10/25 (40.0)	9/28 (32.1)	17/27 (63.0)	13/23 (56.5)	
Metastatic at presentation^‡^	12/29 (41.4)	9/27 (33.3)	12/29 (41.4)	17/30 (56.7)	15/29 (51.7)	
Management						0.133
Resection + primary anastomosis	19 (63.3)	16 (57.1)	20 (62.5)	9 (30.0)	14 (48.3)	
Resection + stoma	4 (13.3)	5 (17.9)	5 (15.6)	9 (30.0)	7 (24.1)	
Non-resectional stoma	0	2 (7.1)	0	7 (23.3)	1 (3.4)	
Colonic stent	0	0	1 (3.1)	0	1 (3.4)	
Non-operative	7 (23.3)	5 (17.9)	6 (18.8)	5 (16.7)	6 (20.7)	
Any stoma at index presentation	4 (13.3)	7 (25.0)	5 (15.6)	16 (53.3)	8 (27.6)	

**Table 3:** Association between calendar year and characteristics of emergency colorectal cancer presentation. ^†^Adjusted odds ratios (aORs) were derived from multivariable logistic regression models including calendar year, age, and sex. Calendar year was modelled as a continuous variable; ORs therefore represent the change in odds associated with each successive calendar year. CI: confidence interval

Outcome	n	Unadjusted OR per year (95% CI)	p-value	Adjusted OR^†^ per year (95% CI)	p-value
Stage IV disease	144	1.20 (0.95-1.51)	0.137	1.20 (0.94-1.52)	0.138
High-grade tumour	127	1.39 (1.07-1.82)	0.015	1.39 (1.07-1.82)	0.015
Metastatic disease at presentation	144	1.20 (0.95-1.51)	0.137	1.20 (0.94-1.52)	0.138
Primary anastomosis	149	0.79 (0.63-1.00)	0.049	0.79 (0.63-1.00)	0.050
Stoma formation at index presentation	149	1.35 (1.03-1.77)	0.027	1.37 (1.04-1.80)	0.026

Tumour stage was available for 144 of 149 patients (96.6%). Stage was unknown in five patients (3.4%) for whom available investigations were insufficient to permit reliable stage assignment. Among patients with known stage, stage IV disease was present in 41.4% (12/29) in 2019, 33.3% (9/27) in 2020, 41.4% (12/29) in 2021, 56.7% (17/30) in 2022, and 51.7% (15/29) in 2023. Although stage IV disease was more frequent in later study years, there was no statistically significant temporal association in either unadjusted analysis (OR 1.20 per year, 95% CI 0.95-1.51; p = 0.137) or following adjustment for age and sex (aOR 1.20, 95% CI 0.94-1.52; p = 0.138).

Tumour grade was available for 127 patients. High-grade tumours accounted for 29.2% (7/24) of cases with known grade in 2019, increasing to 63.0% (17/27) in 2022 and 56.5% (13/23) in 2023. Increasing calendar year was significantly associated with high-grade disease (OR 1.39 per year, 95% CI 1.07-1.82; p = 0.015). This association was unchanged following adjustment for age and sex (aOR 1.39, 95% CI 1.07-1.82; p = 0.015).

Metastatic status at presentation was available for 144 of 149 patients (96.6%), with metastatic status unknown in five patients for whom available investigations were insufficient to determine the presence or absence of distant metastatic disease. As expected, the annual distribution of metastatic disease corresponded with that of stage IV disease: 41.4% (12/29) in 2019, 33.3% (9/27) in 2020, 41.4% (12/29) in 2021, 56.7% (17/30) in 2022, and 51.7% (15/29) in 2023. There was no statistically significant temporal association in either unadjusted (OR 1.20 per year, 95% CI 0.95-1.51; p = 0.137) or age- and sex-adjusted (aOR 1.20, 95% CI 0.94-1.52; p = 0.138) analysis.

Management and temporal trends

Management strategies varied descriptively across the study period, although the overall distribution did not differ significantly between years (p = 0.133). Across the entire cohort, 78 patients underwent resection with primary anastomosis, 30 underwent resection with stoma formation, 10 underwent non-resectional stoma formation, two were managed with colonic stenting, and 29 were managed non-operatively. In 2022, 16 of 30 patients (53.3%) underwent stoma formation at the index presentation, comprising nine patients undergoing resection with stoma formation and seven undergoing non-resectional stoma formation. The annual management data underlying these figures are shown in Table 2. Temporal trends in selected tumour characteristics and management outcomes were assessed using logistic regression, with calendar year modelled as a continuous variable (Table 3).

The proportion of patients undergoing primary anastomosis decreased from 63.3% (19/30) in 2019 to 30.0% (9/30) in 2022, before increasing to 48.3% (14/29) in 2023. Increasing calendar year was associated with reduced odds of primary anastomosis on unadjusted analysis (OR 0.79 per year, 95% CI 0.63-1.00; p = 0.049). Following adjustment for age and sex, the association did not meet the prespecified threshold for statistical significance (aOR 0.79, 95% CI 0.63-1.00; p = 0.050).

Stoma formation during the index emergency presentation occurred in 13.3% (4/30) of patients in 2019, 25.0% (7/28) in 2020, 15.6% (5/32) in 2021, 53.3% (16/30) in 2022, and 27.6% (8/29) in 2023. Increasing calendar year was significantly associated with stoma formation (OR 1.35 per year, 95% CI 1.03-1.77; p = 0.027), and this association persisted following adjustment for age and sex (aOR 1.37, 95% CI 1.04-1.80; p = 0.026).

Screening history

Documented prior engagement with CRC screening was uncommon among patients presenting emergently. Prior colonoscopy was documented in 7.4% of patients, whilst 7.1% had documented previous participation in the NBCSP. Screening history was not available for all patients.

## Discussion

In this regional Australian cohort, emergency CRC presentations did not increase during the COVID-19 restriction period. Absolute emergency presentation volumes remained stable across the complete calendar years from 2019 to 2022, ranging from 28 to 32 cases annually. Emergency presentations also remained stable relative to elective CRC surgical activity, accounting for approximately 15% before and during the COVID-19 restriction period. However, secondary analyses identified significant temporal increases in high-grade disease and stoma formation at index presentation, whilst stage IV and metastatic disease were more frequent in later years but did not demonstrate statistically significant temporal trends. These findings suggest that stability in emergency presentation frequency may not fully capture changes in the clinical and oncological characteristics of emergency CRC.

The stability in emergency presentation volumes contrasts with reports from several international settings, where pandemic-related disruption to screening and diagnostic pathways was associated with increased emergency presentation and concern regarding more advanced disease at diagnosis [1,3,8,9]. Differences in the extent of pandemic-related healthcare disruption may partly explain these findings. Queensland experienced relatively low levels of community transmission during much of the early pandemic and avoided the prolonged restrictions experienced in some Australian and international jurisdictions [10]. Consequently, disruption to primary care and diagnostic and cancer treatment pathways may have been less pronounced in this regional Queensland population, potentially mitigating some of the downstream effects reported elsewhere [11]. These findings should therefore not be assumed to be directly generalisable to health systems that experienced more prolonged or severe disruption.

Despite stable presentation volumes, high-grade disease became significantly more likely with successive calendar years, independent of age and sex. Stage IV and metastatic disease were more frequent descriptively in 2022 and early 2023; however, neither demonstrated a statistically significant temporal association. Conversely, the distribution of acute presentation type remained broadly unchanged. These findings provide evidence of a temporal change in tumour grade but do not establish that COVID-19 was responsible. The retrospective design, modest cohort size, and inclusion of an early post-restriction period preclude causal inference, and larger studies are required to determine whether this represents a reproducible change in tumour characteristics.

Stoma formation at the index emergency presentation also increased significantly over time; however, this association was strongly influenced by a marked increase in 2022, when 53.3% of patients underwent stoma formation, including seven non-resectional stomas. The distribution of acute presentation type did not differ significantly across study years, and bowel obstruction was not more frequent in 2022 than in preceding years, suggesting that the increase was not simply attributable to a greater proportion of obstructing presentations. Stoma formation included both resection with stoma and non-resectional diversion for decompression, palliation, or subsequent oncological treatment and should therefore not be considered a direct surrogate for disease severity. No specific local policy or practice change was identified to explain the 2022 increase. Given the small annual cohorts and subsequent reduction in stoma formation in 2023, the temporal association should be interpreted cautiously and may reflect differences in case mix, operative decision-making, or random variation.

Documented prior screening engagement was uncommon among emergency presenters, with only 7.4% having a documented prior colonoscopy and 7.1% documented participation in the NBCSP. This suggests that changes in screening activity alone may have had limited influence on emergency presentation frequency within this cohort, although incomplete documentation of screening history limits definitive conclusions. Importantly, the low level of documented prior screening engagement also suggests that screening alone is unlikely to prevent all emergency CRC presentations and highlights the importance of accessible diagnostic pathways for symptomatic patients.

The study was prompted by a clinical perception that emergency presentations of advanced CRC had increased during the pandemic. Whilst this was not supported in terms of presentation frequency, the observed increase in high-grade disease and the marked increase in stoma formation in 2022 suggest clinicians may have encountered greater complexity without an increase in overall case numbers. This highlights the importance of evaluating tumour characteristics and management alongside presentation frequency when assessing the effects of healthcare disruption.

This study has several limitations. Its retrospective, single-health-service design and modest sample size limit statistical power and generalisability. Elective CRC resections were used as the comparator for the relative emergency presentation analysis and do not represent the total underlying incidence of CRC; however, absolute emergency presentation volumes were also examined and remained stable across the complete calendar years studied. Tumour stage and metastatic status were unavailable in five patients because of incomplete staging investigations, which may have introduced selection bias into analyses of advanced and metastatic disease. Grouping 2020-2022 as a single COVID-19 restriction period may obscure variation between different phases of the pandemic, although annual data were also examined separately. The marked increase in stoma formation in 2022 substantially influenced the observed temporal association and could not be attributed to a documented local policy or practice change. Finally, the regional Queensland setting, where the timing and extent of healthcare disruption differed from more heavily affected Australian and international jurisdictions, limits extrapolation of these findings to more severely affected health systems.

More broadly, the COVID-19 pandemic provides an example of how disruption to primary care, diagnostic services, and endoscopic capacity may affect cancer pathways. Although this study cannot establish a causal relationship between such disruption and the observed temporal changes, the implications extend beyond COVID-19 to future pandemics and other periods of health-system strain. Maintaining access to population screening, timely assessment of symptoms, and diagnostic investigation should remain a priority during periods of healthcare disruption. The low level of documented prior screening among emergency presenters in this cohort further reinforces the need to preserve both preventative services and accessible pathways for investigation of symptomatic patients.

## Conclusions

Emergency CRC presentation volumes remained stable during the COVID-19 restriction period within this regional Australian health service, alongside relatively preserved elective CRC surgical activity. There was also no significant increase in emergency presentations relative to elective surgical activity. Increasing calendar year was independently associated with high-grade disease and stoma formation, although the stoma association was influenced by a marked increase in 2022 and should be interpreted cautiously. Stage IV and metastatic disease were more frequent in later years but did not demonstrate statistically significant temporal trends. These findings suggest that the clinical perception prompting this study may have reflected changes in the characteristics and management complexity of emergency presentations rather than an increase in their overall frequency.

More broadly, the COVID-19 pandemic provides an example of how disruption to primary care, diagnostic services, and endoscopic capacity may affect cancer pathways. Preserving access to screening, timely assessment of symptoms, and diagnostic investigation should therefore remain a priority during future pandemics and other periods of healthcare disruption. However, the low level of documented prior screening engagement among patients presenting emergently in this cohort highlights that screening alone is unlikely to prevent all emergency CRC presentations, reinforcing the importance of maintaining accessible pathways for both preventative care and symptomatic investigation.
